# Sibling empathy among preschoolers in China: analyzing emotional responses and family influences

**DOI:** 10.3389/fpsyg.2025.1546521

**Published:** 2025-06-06

**Authors:** Xiaolu Ye, Nor Aniza Ahmad, Nur Aimi Nasuha Binti Burhanuddin, Ruohan Xie, Meng Na

**Affiliations:** ^1^Department of Student Affairs, Zhejiang Industry & Trade Vocational College, Wenzhou, Zhejiang, China; ^2^Department of Foundations of Education, Faculty of Educational Studies, Universiti Putra Malaysia, Serdang, Malaysia; ^3^Zhejiang Normal University, Hangzhou, China; ^4^School of Education, Wenzhou University, Wenzhou, Zhejiang, China; ^5^Graduate School of Business, Universiti Kebangsaan Malaysia, Bangi, Malaysia

**Keywords:** sibling empathy, preschool children, general empathy, sibling relationships, emotional development, socio-emotional development, birth order, gender differences

## Abstract

**Introduction:**

Empathy between siblings plays a pivotal role in early socio-emotional development, yet limited research has explored this construct within the context of Chinese preschool-aged children, particularly in light of China’s changing family structures. This study addresses this gap by examining the characteristics of sibling empathy and its associations with general empathy and sibling relationship quality.

**Methods:**

A total of 222 children aged 3 to 6 years from two-child families in Zhejiang Province, China, participated in this study. Sibling empathy was assessed using the newly developed Measurement of Sibling Empathy in Chinese Preschool Children (MSCP). The study examined differences in sibling empathy across age, gender, birth order, and sibling gender combinations (i.e., two boys, two girls, and one boy and one girl). A mediation model was tested to evaluate the role of sibling empathy in linking general empathy to sibling relationship quality.

**Results:**

Analysis revealed that younger children exhibited significantly lower empathy for sadness, and second-born children showed higher empathy for fear. Two-girl sibling pairs demonstrated greater empathy for anger than mixed-gender pairs. No significant gender differences were observed. General empathy was positively associated with both sibling empathy and sibling relationship quality. Structural Equation Modeling (SEM) indicated that sibling empathy significantly mediated the relationship between general empathy and sibling relationship quality.

**Discussion:**

The findings contribute to developmental and cultural theories of empathy by highlighting emotion-specific variations in sibling empathy and their implications for sibling dynamics in Chinese families. While the cross-sectional design and reliance on mother-reported data pose limitations, this study offers foundational insights and points toward targeted interventions to foster empathy and improve sibling relationships in early childhood.

## 1 Introduction

Sibling relationships are a fundamental aspect of early childhood development, providing a unique context for emotional and social learning. One critical component of these relationships is sibling empathy, a psychological phenomenon where genetically related children share feelings and thoughts due to their shared biological parents (Jambon et al., 2019; Qian et al., 2022; Walęcka-Matyja, 2017). Research has increasingly shown that the presence of siblings significantly influences children’s empathy development, confirming the existence and importance of sibling empathy (Jambon et al., 2019). This phenomenon can be analyzed through the lens of Inner Group Biases, where sibling empathy represents a form of empathy manifested within a specific group, namely siblings (McLoughlin and Over, 2017; Over, 2018). Additionally, the empathy-altruism hypothesis suggests that empathy is crucial for promoting and maintaining positive sibling relationships (Walęcka-Matyja, 2017; Persson and Shrivastava, 2016; Schroeder et al., 2015).

The implementation of China’s “two-child policy” in 2016, which replaced the long-standing “one-child policy,” has brought significant changes to family dynamics (Chen et al., 2017). This policy shift has led to the emergence of siblings in many families, increasing the proportion of two-child households from 30% to 50% within 3 years (National Bureau of Statistics of China, 2019). This demographic change underscores the necessity of understanding sibling relationships, including sibling empathy (Noten et al., 2020; Shao et al., 2022). Despite the policy’s profound impact, there remains a dearth of empirical research on sibling empathy among Chinese children, with only 15% of recent studies focusing on sibling interactions in the Chinese context (Bin, 2023). This gap highlights the importance of exploring sibling empathy, especially in light of the unique cultural and familial structures in China, making this an important and timely area of exploration.

Empathy is a multifaceted psychological process where individuals perceive or imagine others’ emotions and experience their feelings (Jolliffe and Farrington, 2006; Pan et al., 2013; Singer and Lamm, 2009). It is a critical component of socio-emotional development in children (Sallquist et al., 2009). Given that siblings are natural playmates and spend considerable time together, they significantly influence each other’s emotional development (Qian et al., 2022; Kim et al., 2006; Pérez, 2013). The concept of group empathy, influenced by Inner Group Biases, suggests that empathy manifests differently depending on the context and target, such as sibling empathy, which develops early in life (McLoughlin and Over, 2017; Over, 2018; Baron and Banaji, 2006; Hamlin et al., 2013).

Hoffman’s Theory of Empathy Development posits that empathy evolves throughout the lifespan, with unique characteristics emerging during the preschool years (Hoffman, 1996; Chan et al., 2023). In preschool children, empathy primarily involves affective empathy—vicariously experiencing others’ emotions—and cognitive empathy—understanding others’ emotions (Decety and Holvoet, 2021; Heyes, 2018; Barata et al., 2024). While affective empathy develops early and remains stable during the preschool years (Bensalah et al., 2016; Howe, 2008), cognitive empathy undergoes significant development during this period (Decety et al., 2015; Brink et al., 2011).

Existing measures of empathy in preschool children, such as the Griffith Empathy Measure (GEM) (Dadds et al., 2008), the Empathy Continuum (Strayer, 1993), and the Virtual Reality Empathy Test (VRET) (Kim et al., 2024), provide valuable tools for assessing empathy. Some researchers argue that empathy also includes behavioral responses to interpersonal interactions, such as social skills or prosocial behaviors (Chen et al., 2017; Lawrence et al., 2004; Stern et al., 2015). Measures like the Empathy Questionnaire (EmQue) (Rieffe et al., 2010) and the Measure of Empathy in Early Childhood (MEEC) (Chan et al., 2023; Levantini et al., 2024) reflect this broader perspective. The empathy-altruism hypothesis asserts that empathetic feelings drive altruistic motivation, fostering prosocial behaviors and positive relationships (Persson and Shrivastava, 2016; Schroeder et al., 2015). However, empathy is not the sole factor influencing prosocial behavior; Theory of Mind (ToM), personality, and emotion regulation abilities also play significant roles (Brown et al., 2016; Cristofani et al., 2020; Simon and Nader-Grosbois, 2023).

In China, the Empathy Continuum (Strayer, 1993) is widely used to measure empathy in preschool children. This oral-report task integrates affective sharing and cognitive attribution of emotions and has demonstrated good applicability in China, making it suitable for adapting to measure sibling empathy among Chinese preschool children (Shao et al., 2022; Yan et al., 2019).

Given the importance of sibling relationships in early childhood and the unique cultural context of China, this study aims to explore the characteristics of sibling empathy among preschool children in China. Specifically, it seeks to address the following research questions:

1. Are there gender and age differences in sibling empathy among Chinese preschool children?
2. Are there birth order and gender combination differences in sibling empathy?
3. What is the association between general empathy and sibling empathy?
4. Does sibling empathy mediate the relationship between general empathy and sibling relationship quality?

By examining these questions, this study aims to deepen our understanding of the dynamics of sibling empathy and its impact on sibling relationships, offering a nuanced perspective that accounts for cultural context and developmental stages. This research is crucial as it fills a significant gap in the existing literature, where less than 20% of studies on child development have explored sibling interactions in non-Western settings (Liu and Fisher, 2022). Furthermore, understanding these dynamics can inform parenting practices and educational strategies, potentially benefiting over 200 million children living in two-child households in China (National Bureau of Statistics of China, 2019). Ultimately, this study provides a comprehensive framework for future research, paving the way for interventions that promote healthier sibling relationships and overall socio-emotional development in children.

## 2 Literature review

### 2.1 Theoretical understanding of empathy

The theoretical foundation of this study is primarily anchored in Hoffman’s Theory of Empathy Development and Group Empathy Theory, providing a comprehensive framework for understanding the development and significance of sibling empathy in early childhood. These theories elucidate the processes through which empathy emerges and evolves, particularly within the context of sibling relationships.

Hoffman’s Theory of Empathy Development (Hoffman, 1996) offers a nuanced understanding of how empathy progresses through different stages across the lifespan. According to Hoffman, empathy begins as a primarily affective response in infancy, where children vicariously experience the emotions of others. As they grow, cognitive empathy—the ability to understand and predict others’ emotional states—becomes more prominent. This theory is particularly relevant for this study as it highlights the developmental trajectory of empathy from early childhood onwards (Hughes et al., 1981). In the context of sibling relationships, Hoffman’s theory suggests that the frequent and intimate interactions between siblings provide a rich environment for practicing and refining empathic skills. The shared experiences and emotional exchanges between siblings serve as a training ground for both affective and cognitive empathy, thereby facilitating their overall socio-emotional development (Decety et al., 2015; Brink et al., 2011; Aldridge et al., 2017).

Group Empathy Theory, as explored by Baron and Banaji (2006) and further developed by McLoughlin and Over (2017), posits that empathy is more robust within in-groups due to shared identities and experiences. This theory is particularly pertinent in the context of sibling relationships, where genetic and familial bonds naturally create an in-group. Sibling empathy can thus be viewed as a specific manifestation of group empathy, where the familial bond enhances empathic responses. This theory suggests that the empathy siblings feel for each other is intensified by their shared family environment and frequent interactions. The strong emotional bonds between siblings foster a deeper understanding and concern for each other’s well-being, which is essential for maintaining positive sibling relationships (Over, 2018).

Combining Hoffman’s Theory of Empathy Development and Group Empathy Theory provides a comprehensive framework for understanding sibling empathy. Hoffman’s theory explains the developmental trajectory of empathy, while Group Empathy Theory contextualizes this development within the specific social structure of sibling relationships. Together, these theories elucidate how empathy evolves in early childhood and how sibling interactions specifically contribute to this process.

In addition to these theories, it is essential to consider the socio-cultural context, particularly the concept of familism prevalent in Chinese culture. Familism emphasizes family cohesion, respect for authority, and interdependence among family members (Lu et al., 2023). This cultural backdrop provides a unique lens through which the development of sibling empathy can be understood, highlighting the importance of cultural norms and values in shaping empathic behaviors (Feshbach, 1975; Bowie et al., 2013).

**Figure 1:**
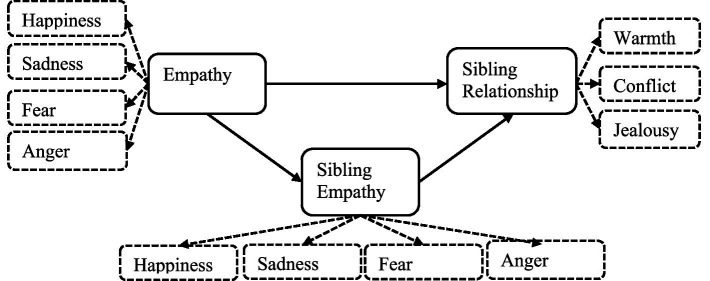
Research framework.

By integrating Hoffman’s Theory of Empathy Development and Group Empathy Theory (refer to Figure 1), this study aims to explore the characteristics of sibling empathy among Chinese preschool children. This theoretical framework not only provides a basis for understanding the mechanisms underlying sibling empathy but also offers insights into how these processes can be supported and enhanced within the specific cultural context of China.

### 2.2 Hypothesis development

Sibling empathy in preschool children is a nuanced and emerging field with varying findings regarding age and gender influences. Some studies indicate that empathy in preschool children increases with age, suggesting developmental maturation enhances empathetic capacities (Rieffe et al., 2010; Luo et al., 2019; Zhang et al., 2019; Zhai et al., 2023). Conversely, other research posits that empathy development may be age-independent or even negatively correlated with age (Chen et al., 2017; Anastassiou-Hadjicharalambous and Warden, 2007; Eisenberg-Berg and Mussen, 1978). Gender differences in empathy development are also debated. Some research suggests that girls develop empathy faster than boys during preschool years (Simon and Nader-Grosbois, 2023; Zhang et al., 2019; Zhai et al., 2023), while other studies find no significant gender differences (Luo et al., 2019). Given these conflicting results, it is essential to investigate both age and gender differences in sibling empathy among Chinese preschool children. Therefore, we hypothesize:

*H1:* There are significant gender and age differences in sibling empathy among Chinese preschool children.

Sibling relationships are intricate, characterized by various interactions and influenced by structural features like gender combination and birth order (Zhao and Yu, 2017; Cicirelli, 1995). Gender combination within sibling pairs can significantly impact the quality of sibling relationships. For instance, some studies report increased aggression among same-gender siblings (Minnett et al., 1983), while others find higher intimacy in these pairs (Kier and Lewis, 1998; McHale et al., 2009). Birth order is another critical factor; Western studies typically show no impact on empathy by birth order (Gordon, 2012; Smith et al., 2014; Tucker et al., 2017). However, in the Chinese context, both first-born and second-born children in two-child families exhibit better empathy than only children (Qian et al., 2022; Zhang et al., 2019). These variations highlight the need to examine how gender combination and birth order affect sibling empathy among Chinese preschool children. Thus, we propose:

*H2:* There are significant differences in sibling empathy based on birth order and gender combination among Chinese preschool children.

Emerging research suggests that general empathy can predict sibling empathy. Cross-sectional studies indicate that empathetic younger siblings may foster similar tendencies in older siblings (Dunn and Plomin, 1991). Longitudinal studies further support that siblings’ empathetic concern predicts each other’s empathy over time (Jambon et al., 2019; Sallquist et al., 2009; Yan et al., 2019; Eisenberg et al., 2013). In the Chinese context, individual intrinsic factors like children’s empathy significantly influence sibling empathy (Qian et al., 2022). Given this predictive relationship, we hypothesize:

*H3:* There is a positive association between general empathy and sibling empathy among Chinese preschool children.

The relationship between sibling empathy and sibling relationship quality is crucial for understanding family dynamics. Research shows that expressing empathy within a group is essential for reconciliation during conflicts (Čehajić-Clancy et al., 2016). High levels of sibling empathy strongly predict maintaining close and positive sibling relationships (Kardos et al., 2017). Warm sibling relationships correlate with higher empathy and a better ability to understand others’ perspectives (Zhang et al., 2019; Feinberg, 2003; Zhiqiang et al., 2019). These findings lead us to hypothesize:

*H4:* There is a positive association between sibling empathy and sibling relationship quality among Chinese preschool children.

Lastly, the potential mediating role of sibling empathy in the relationship between general empathy and sibling relationship quality warrants exploration. Studies suggest that empathy enhances sibling warmth over time (Lam et al., 2012; Yu-chuan et al., 2022), and second-born siblings’ empathy significantly impacts sibling warmth more than first-born siblings (Tucker et al., 2017). In Chinese high school students, empathy mediates the relationship between parental differential treatment and sibling relationships (Ze and Jiaqi, 2022; Yang et al., 2024). Therefore, it is plausible that sibling empathy mediates the relationship between general empathy and sibling relationship quality among preschool children. We hypothesize:

*H5:* Sibling empathy mediates the relationship between general empathy and sibling relationship quality among Chinese preschool children.

## 3 Method

### 3.1 Participants

A total of 222 Chinese preschool children (Mage = 5.3 years, SD = 0.80; 49.5% boys), aged between 3 and 6 years, participated in this study. All children came from two-child nuclear families residing in Zhejiang Province, China. The sample included both first-born and second-born children, with an average sibling age gap of 1.6 years (SD = 3.68). Sibling gender composition comprised 23.4% two-boy dyads, 23.4% two-girl dyads, and 53.2% mixed-gender dyads (one boy and one girl).

Participants were recruited through purposive sampling via online parenting forums and preschool networks. Eligibility criteria included: (1) having two co-residing biological children aged 3–6 years, (2) a complete nuclear family structure, and (3) fluency in Mandarin Chinese. Children with diagnosed cognitive impairments or language delays were excluded.

The sample largely represented urban, middle-class households, with 89% of parents holding a university degree or higher. While this cohort provides important insight into sibling dynamics among contemporary Chinese families, its socioeconomic and geographic homogeneity may limit generalizability to rural, lower-income, or ethnically diverse populations. Informed parental consent and child assent were obtained prior to data collection. Attrition was minimal (n = 8), attributed primarily to scheduling conflicts or lack of continued interest.

### 3.2 Measures

#### 3.2.1 The measurement of sibling empathy in Chinese preschool children (MSCP)

The MSCP was developed based on the Empathy Continuum Scoring Manual (Strayer et al., 1992). We received permission from Dr. Laurie Kramer, Professor Emeritus at the University of Illinois, to use the PEPC-SRQ in our research. This tool, along with its scoring system, was adapted to suit the cultural context of Chinese families. Dr. Kramer provided detailed adaptation strategy of PEPC-SRQ specifically adapted for Chinese parents of siblings. We also obtained approval to translate the instrument into Chinese, which we did for the purposes of this study. This involved presenting children with a series of narratives about emotion-evoking situations and asking how they felt after each narrative. The principal character in these narratives was the child’s sibling.

Following multiple interviews with parents from Chinese two-child families, four narratives depicting situations involving four emotions (happiness, sadness, fear, and anger) were created. These narratives were recorded as audio clips, each approximately 15 s long. The themes for each emotion were as follows:

1. *Happiness (Birthday Party)*: “Your sibling’s birthday is coming up. How he/she has been looking forward to this day! At his/her birthday party, family members and friends come to celebrate his/her birthday and give him/her many toys.”
2. *Sadness (Toy Lost)*: “Your sibling has a favorite toy. Wherever he/she goes, he/she always takes it with him/her. Today, however, he/she cannot find the toy if he/she does not know where it is. Your sibling feels he/she has lost his/her toy forever.”
3. *Fear (Sibling Lost)*: “When your sibling wakes up and finds no one at home, he/she goes out to look for family. He/she walked a long way but did not find them. It’s getting dark and he/she wants to go home, but he/she does not know how to get home.”
4. *Anger (Lollipop Snatcher)*: “Your sibling brought a delicious lollipop that he/she absolutely loves. A kid in the neighborhood saw his/her lollipop, then came over and took his/her lollipop and ate it all.”

**Figure 2:**
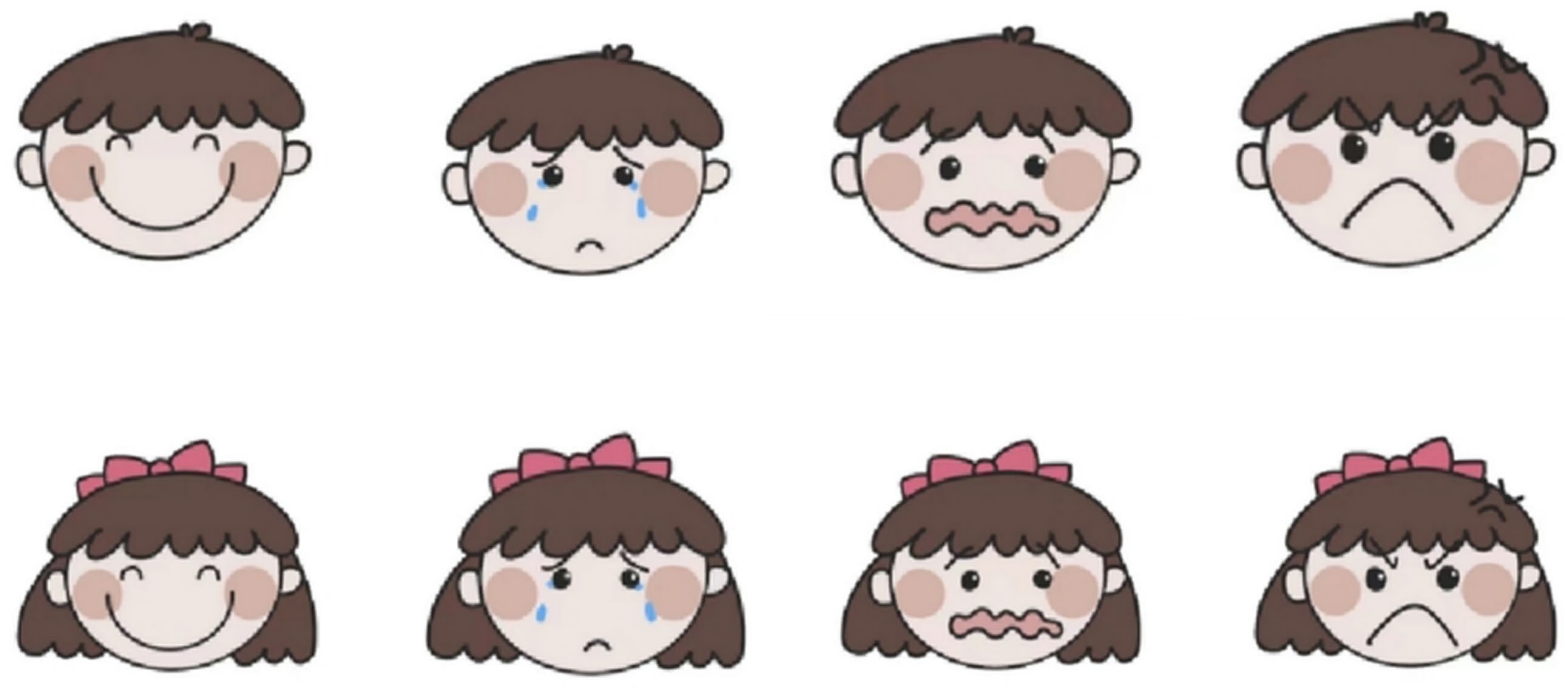
Four emotions pictures represented brother or sister sibling in this study.

**Figure 3:**
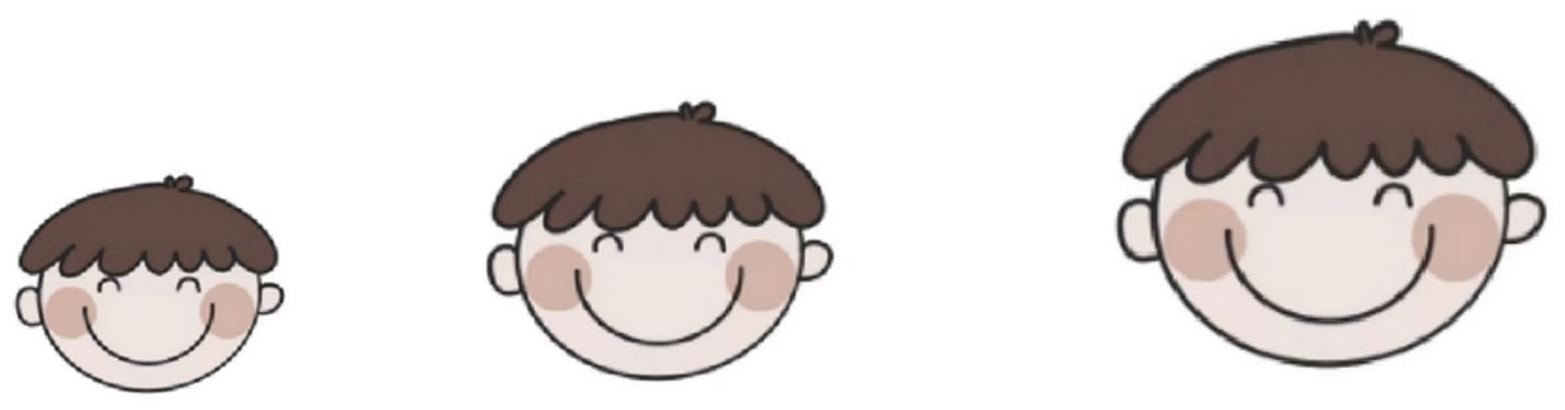
Three picture sizes for emotional intensity.

Given preschool children’s limited expressive language skills, they were shown four pictures of facial expressions (happiness, sadness, fear, and anger) to help them express their understanding of the emotions reflected in the narratives (see Figures 1–3). The Empathy Continuum (EC) Scoring Manual (Strayer et al., 1992) was used to score the children’s responses (see Table 1). In the current sample, scores ranged from 1 to 15 for happiness (M = 4.77, SD = 2.99), 0 to 12 for sadness (M = 2.43, SD = 2.67), 0 to 13 for fear (M = 2.74, SD = 4.11), and 0 to 16 for anger (M = 2.74, SD = 3.60).

**Table 1:** The Empathy Continuum (EC) scoring system in this study.

EC score	EC level (cognitive attribution)	Affect match	Description
0	0	0	No emotion for self (S) OR inaccurate emotion for character (C)
1	0	1	Accurate character’s emotion, but no discordant emotion for self
*No concordant emotions at this level*			
2	II	1	S and C: similar emotion
3		2	S and C: same emotion, different intensity
4		3	S and C: same emotion, same intensity
*No attribution, or irrelevant attribution for own emotion:* “I just did not like it.”			
5	III	1	S and C: similar emotion
6		2	S and C: same emotion, different intensity
7		3	S and C: same emotion, same intensity
*Attribution based on events only:* “I was scared of the dark.”			
8	IV	1	S and C: similar emotion
9		2	S and C: same emotion, different intensity
10		3	S and C: same emotion, same intensity
*Attribution refers to the character’s specific situation:* “I was scared when my brother did not come home after dark.”			
11	V	1	S and C: similar emotion
12		2	S and C: same emotion, different intensity
13		3	S and C: same emotion, same intensity
*Attribution indicates transposition of self into situation and/or association with own experience:* “Scared – There’s no way I’m not going home after dark.”			
14	VI	1	S and C: similar emotion
15		2	S and C: same emotion, different intensity
16		3	S and C: same emotion, same intensity
*Attribution indicates responsiveness to character’s internal state (feelings, thoughts) or general life situation:* “I was sad because my brother must have been terrified.”			
17	VII	1	S and C: similar emotion
18		2	S and C: same emotion, different intensity
19		3	S and C: same emotion, same intensity
*Attribution indicates semantically explicit role taking:* “If I were her, I’d feel just as angry at him.”			

### 3.3 Strengths and difficulties questionnaire

The Empathy Test for 3- to 6-year-olds (Zhai et al., 2023) and the Questionnaire of Children’s Sibling Relationship Quality in Early Childhood (Li and Liu, 2020) were selected as components of the Strengths and Difficulties Questionnaire (Goodman, 2001). Empathy was measured using the Situational Story Test on Empathy for 3- to 6-year-olds (Zhai et al., 2023), which involved presenting a child with a series of narratives about emotion-evoking situations and asking how the child feels after each narrative. The responses were scored using the Empathy Continuum (EC) Scoring Manual (Strayer et al., 1992).

The sibling relationship was measured using the Questionnaire of Children’s Sibling Relationship Quality in Early Childhood (Aged 0–8) (Li and Liu, 2020; Xiaoting, 2018), which consists of 18 items that assess children’s sibling relationships in various daily situations. Mothers rated warmth, conflict, and jealousy toward the sibling relationship using a 5-point scale. The Chinese versions of these tests have shown good psychometric properties (Qian et al., 2022; Shao et al., 2022; Liu and Fisher, 2022; Zhang et al., 2019; Zhai et al., 2023). In this study, Cronbach’s alphas for empathy and sibling relationship were 0.76, 0.71, and 0.83, respectively.

### 3.4 Procedure

Each child participated in an individual video session with a trained researcher in a quiet room at home. The researcher explained that the child would listen to a story and then answer a series of questions. Four audio-recorded, emotion-eliciting sibling stories were presented in randomized order. Each story was tailored to the gender and birth order of the participant’s sibling (younger brother, younger sister, older brother, or older sister).

Prior to story presentation, the researcher confirmed that the child could correctly identify basic emotions using pictorial aids representing happiness, sadness, fear, anger, and indifference. “Indifference” was introduced as “not feeling anything special or strong,” and explained using a neutral facial expression and simple phrases such as “like you do not care much.” To help children evaluate emotional intensity, three image sizes were used: small (mild), medium (moderate), and large (severe), reinforcing the scale visually.

After each story, the following questions were asked:

1. “Do you think the younger/older brother/sister in the story feels happiness, sadness, anger, fear, or indifference?”
2. “Is his/her feeling mild, moderate, or severe?”
3. “How do you feel after hearing the story? Happiness, sadness, anger, fear, or indifference?”
4. “Is your feeling mild, moderate, or severe?”
5. “Why do you feel that way?”

During questioning, children viewed the corresponding facial expression images. If multiple emotions were mentioned, they were prompted to select the strongest one. All responses were video-recorded and later coded using the Empathy Continuum (EC) Scoring Manual (Strayer et al., 1992).

When both siblings in a family participated, the younger sibling was always assessed first to reduce social desirability and imitation effects. This decision was grounded in developmental research suggesting that younger children are more susceptible to modeling older siblings’ behaviors.

After the interview session, the child and their mother completed the Empathy Test for 3- to 6-year-olds and the Questionnaire of Children’s Sibling Relationship Quality in Early Childhood, respectively, with guidance from the researcher. To evaluate test–retest reliability, the same measures were re-administered after an eight-week interval. All participation was voluntary, anonymous, and conducted via a secure online platform.

### 3.5 Analysis

SPSS (Version 27) and Mplus (Version 8) were used for data analysis. The analysis was conducted without missing data. First, reliability was evaluated using Kendall’s Wa and test–retest correlation with Pearson’s correlations. Values of Kendall’s Wa greater than 0.90 were considered acceptable (Liebezeit et al., 2007; Ahmed et al., 2024; Ahmed et al., 2022). Second, ANOVA was performed to evaluate age, gender, birth order, and gender combination differences. Third, Pearson’s correlations between sibling empathy, overall empathy, and sibling relationship were examined. Finally, SEM was used to test the latent variable mediation model. Bootstrap confidence intervals (CIs) were applied to determine whether the mediating effect was significant. If the CI did not include zero, the effect was considered significant.

## 4 Results

### 4.1 Reliability

In this study, Kendall’s Wa was used to measure rater consistency for the sibling empathy questionnaire. The analysis results show that Kendall’s Wa for each factor is greater than 0.9, indicating strong consistency among raters, as shown in Table 2. Pearson’s correlation was calculated to assess test–retest reliability, and the coefficients were acceptable, confirming the consistency of the questionnaire over time: Happiness = 0.88, Sadness = 0.85, Fear = 0.90, and Anger = 0.80.

**Table 2:** Sibling empathy test—Kendall’s Wa and test–retest correlation.

Variable	Kendall’s Wa	Test–retest correlation
Happiness	0.96	0.88
Sadness	0.93	0.85
Fear	0.92	0.90
Anger	0.96	0.80

### 4.2 Gender and age differences

Since the data did not conform to a normal distribution, the logarithmic conversion method was used for correction. After correction, the data achieved a normal distribution (Skew ±2, Kurtosis ±7), allowing for independent sample t-tests and variance analysis (refer to Table 3).

**Table 3:** Mean and standard deviation for sibling empathy.

Variable	Mean ± SD
Happiness	4.77 ± 2.99
Sadness	2.43 ± 2.67
Fear	2.74 ± 4.11
Anger	2.74 ± 3.60

An independent sample t-test was used to investigate whether the gender of the children affected sibling empathy. The results showed no significant differences in happiness [t(220) = −1.97, *p* = 0.51], sadness [t(220) = 1.28, *p* = 0.20], fear [t(220) = 1.33, *p* = 0.18], and anger [t(220) = −1.00, *p* = 0.32].

Table 4 shows the results of a one-way ANOVA conducted to compare age differences in sibling empathy. The analysis, in which age groups (3–4 years, 4–5 years, and 5–6 years) were the independent variables and sibling empathy was the dependent variable, revealed a significant difference in sibling empathy to sadness at the *p* < 0.05 level [*F*(1, 221) = 4.41*]. The LSD test showed that sibling empathy to sadness in 3- to 4-year-olds was significantly lower than in 4–5 and 5- to 6-year-olds at the *p* < 0.05 level. No significant differences were found for happiness [*F*(1, 221) = 2.99], fear [F(1, 221) = 2.87], and anger [F(1, 221) = 2.17] at the *p* < 0.05 level.

**Table 4:** Results of one-way ANOVA for sibling empathy to sadness by age.

Variables	3–4 Y	4–5 Y	5–6 Y	F	LSD
M ± SD	0.93 ± 0.22	1.01 ± 0.31	1.02 ± 0.13	4.41	3–4 Y < 4–5 Y*
					3–4 Y < 5–6 Y*

*p < 0.05.

### 4.3 Birth order and gender combination

An independent sample t-test was used to investigate whether birth order affected sibling empathy. The results showed a significant difference in fear [t(220) = −3.29, *p* = 0.001*], with no significant differences in happiness [t(220) = −1.01, *p* = 0.32], sadness [t(220) = −0.61, *p* = 0.54], and anger [t(220) = −0.74, *p* = 0.46]. The findings indicated that second-born children exhibited significantly higher sibling empathy for fear than first-born children, as shown in Table 5.

**Table 5:** Results of independent sample *t*-test for sibling empathy to fear by birth order.

Variables	First-born	Second-born	df	*t*	*p*
M ± SD	0.92	1.04	220	−3.29	0.001**

**p < 0.01.

Table 6 shows the results of a one-way ANOVA conducted to compare gender combination differences in sibling empathy. The analysis, with gender combinations (two boys, two girls, and one boy one girl) as the independent variable and sibling empathy as the dependent variable, revealed a significant difference for gender combination in sibling empathy to anger at the *p* < 0.05 level [*F*(1, 221) = 4.45*]. The LSD test showed that sibling empathy to anger in two-girl families was significantly higher than in mixed-gender families at the *p* < 0.05 level. No significant differences were found for happiness [F(1, 221) = 2.11], sadness [F(1, 221) = 1.19], and fear [F(1, 221) = 0.92] at the *p* < 0.05 level.

**Table 6:** Results of one-way ANOVA for sibling empathy to anger by gender combination.

Variables	3–4 Y	4–5 Y	5–6 Y	F	LSD
M ± SD	0.93 ± 0.22	1.01 ± 0.31	1.02 ± 0.13	4.45	One boy one girl < two girls*

*p < 0.05.

### 4.4 Correlations between sibling empathy, empathy, and sibling relationship

The correlation matrix of the variables is shown in Table 7. Results of the correlation analysis indicated that happiness was positively associated with sadness (*r* = 0.51, *p* < 0.01), fear (*r* = 0.49, *p* < 0.01), and anger (*r* = 0.42, *p* < 0.01). Sadness was positively associated with fear (*r* = 0.63, *p* < 0.01) and anger (*r* = 0.60, *p* < 0.01). Fear was positively associated with anger (*r* = 0.63, *p* < 0.01).

**Table 7:** Results of correlations between sibling empathy, empathy, and sibling relationship.

Variable	Happiness	Sadness	Fear	Anger	Empathy	Sibling relationship
Happiness	1					
Sadness	0.51**	1				
Fear	0.49**	0.63**	1			
Anger	0.42**	0.60**	0.63**	1		
Empathy	0.29**	0.42**	0.38**	0.41**	1	
Sibling Relationship	0.25**	0.36**	0.39**	0.32**	0.34**	1

**p < 0.01.

Additionally, empathy was positively associated with sibling empathy for happiness (*r* = 0.29, *p* < 0.01), sadness (*r* = 0.42, *p* < 0.01), fear (*r* = 0.38, *p* < 0.01), and anger (*r* = 0.41, *p* < 0.01). Sibling relationships were positively related to sibling empathy for happiness (*r* = 0.25, *p* < 0.01), sadness (*r* = 0.36, *p* < 0.01), fear (*r* = 0.39, *p* < 0.01), and anger (*r* = 0.32, *p* < 0.01). Moreover, empathy was positively associated with sibling relationships (*r* = 0.34, *p* < 0.01).

#### 4.4.1 The latent variable mediation model

The structural equation model (SEM) demonstrated excellent fit to the data, with CFI = 0.99, TLI = 0.99, IFI = 0.99, RMSEA = 0.02, and SRMR = 0.03 (Table 8), indicating strong model adequacy.

**Table 8:** Results of model fitting index.

Index	Absolute fit	Value added fit
Specific classification	RMSEA	SRMR
Judging standard	< 0.05	≤ 0.10
Fitting result	0.02	0.03

Table 9 presents the results of the regression paths. General empathy significantly predicted sibling empathy (*B* = 0.73, *p* < 0.001), and both empathy (*B* = 0.08, *p* < 0.001) and sibling empathy (*B* = 0.09, *p* < 0.001) significantly predicted sibling relationship quality. All variables were standardized. These findings support the proposed mediation model, in which sibling empathy functions as a conduit through which general empathy enhances sibling relationships.

**Table 9:** Regression analysis of the relationship between empathy, sibling empathy, and sibling relationship.

Outcome variable	Predictive variable	R2	*b*	SE	*t*	*p*	LLCI	ULCI
Sibling empathy		0.29						
	Empathy		0.73	0.13	5.76	0.000	0.54	0.96
Sibling relationship		0.24						
	Empathy		0.08	0.03	3.11	0.002	0.04	0.13
	Sibling empathy		0.09	0.02	4.15	0.000	0.06	0.12

**p < 0.01; ***p < 0.001.

Table 10 further details the mediation pathways. The total indirect effect (*B* = 0.06, *p* < 0.001) accounted for 43.36% of the total effect (*B* = 0.14), while the direct effect (*B* = 0.08, *p* < 0.01) accounted for 56.64%. The 95% confidence interval for the indirect effect did not include zero, confirming its significance. These findings provide robust empirical support for a partial mediation model: general empathy influences sibling relationship quality both directly and indirectly via sibling empathy.

**Table 10:** Testing the pathways of the multiple mediation model.

Effect	Estimate	SE	Lower	Upper	*p*	Ratio of indirect to total effect	Ratio of indirect to direct effect
Total effect	0.14	0.03	0.10	0.19	0.000		
Direct effect	0.08	0.03	0.04	0.13	0.002	56.64%	
Total indirect effect	0.06	0.02	0.04	0.09	0.000	43.36%	76.54%

**p < 0.01; ***p < 0.001.

This model is theoretically grounded in *Hoffman’s Theory of Empathy Development*, which posits that empathy evolves from affective to cognitive forms through social interaction and perspective-taking. In line with this theory, the significant path from general empathy to sibling empathy reflects children’s growing ability to apply generalized empathic understanding within specific relational contexts. Furthermore, the link between sibling empathy and sibling relationship quality is consistent with *Group Empathy Theory*, which suggests that empathy is more salient and potent within in-groups such as families. The stronger empathic engagement observed within sibling dyads reinforces the idea that frequent, emotionally meaningful interactions foster relationship-enhancing empathy.

Thus, the SEM findings not only confirm the hypothesized mediation mechanism but also align conceptually with developmental and social psychological theories. These results underscore the importance of viewing sibling empathy as both a developmental outcome of general empathic capacity and a relational process embedded within close familial bonds.

Results indicate that sibling empathy mediates the influence of empathy on sibling relationships.

**Figure 4:**
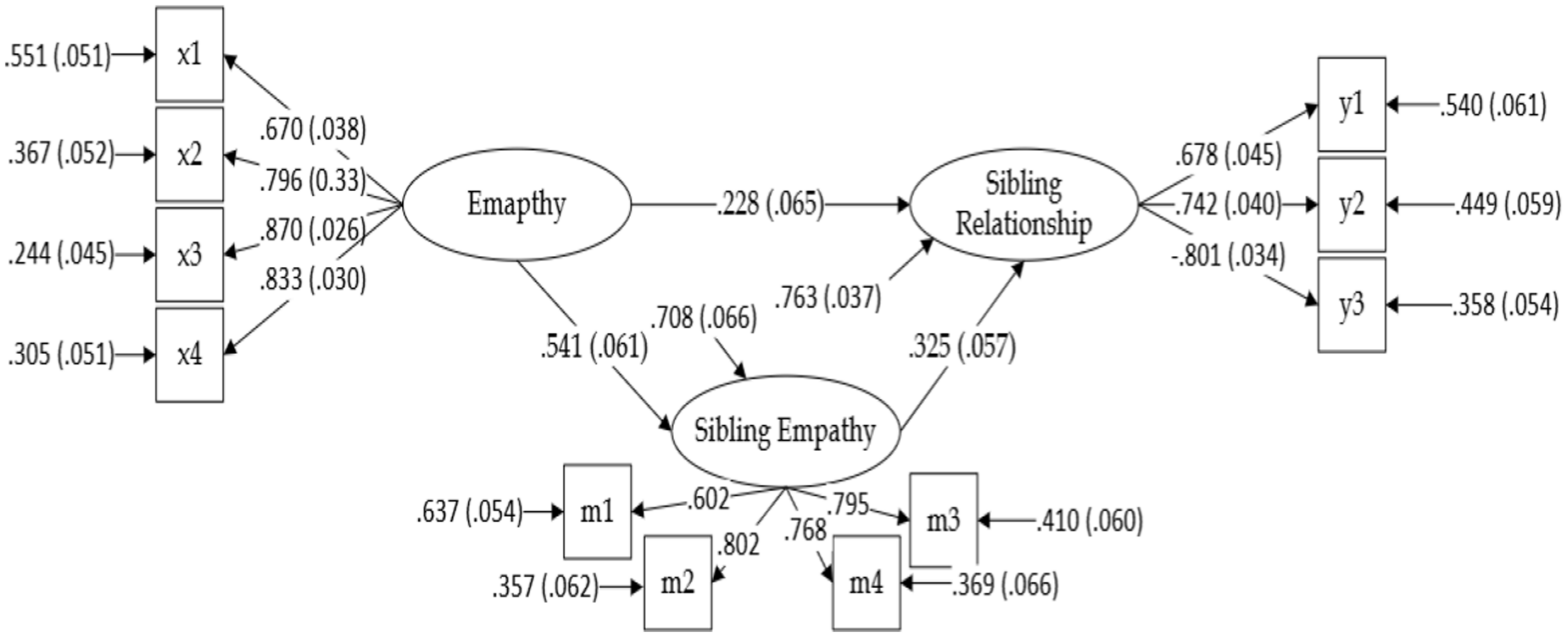
The mediation model. N = 222. The effect of empathy is shown in parentheses. In Mplus 8, the regression coefficient was obtained. x1 → empathy in response to “Happiness” emotion; x2 → empathy in response to “Sadness” emotion; x3 → empathy in response to “Fear” emotion; x4 → empathy in response to “Anger” emotion; m1 → sibling empathy in response to “Happiness” emotion; m2 → sibling empathy in response to “Sadness” emotion; m3 → sibling empathy in response to “Fear” emotion; m4 → sibling empathy in response to “Anger” emotion; y1 → warmth of sibling relationship; y2 → conflict of sibling relationship; y3 → jealousy of sibling relationship.

The mediation model shows the effects of empathy and sibling empathy on sibling relationship (see Figure 4).

## 5 Discussion

Our study is among the first to quantitatively assess sibling empathy in Chinese preschool children, evaluating the psychometric properties of the MSCP. Kendall’s Wa indicated strong inter-rater reliability, while test–retest reliability was within the acceptable range, demonstrating stability over time.

First, the results showed (refer to Table 11) that sibling empathy scores were highest in response to the “happiness” emotion (M = 4.77, SD = 2.99), compared to “sadness” (M = 2.43, SD = 2.67), “fear” (M = 2.74, SD = 4.11), and “anger” (M = 2.74, SD = 3.60). This aligns with previous research on empathy in Chinese preschool children (Zhai et al., 2023) and reflects the general characteristic that the extent of empathy varies depending on the emotion (Olderbak et al., 2014). According to Hoffman’s Theory of Empathy Development, cognitive empathy begins to develop in preschool childhood (Decety et al., 2015; Brink et al., 2011; Lina, 2019). Our results support this, showing cognitive empathy responses primarily at the level of attribution based on events. The large standard deviation indicates that sibling empathy in Chinese preschool children is a less stable psychological trait, subject to environmental fluctuations (Martínez and Nishiyama, 2019).

**Table 11:** Major findings of this study.

Hypothesis	Analysis method	Major findings	Statistical evidence
H1	ANOVA, Independent sample t-test	No significant gender differences in sibling empathy for happiness, sadness, fear, and anger. Significant age differences for sadness, with younger children showing lower empathy.	Happiness: [t(220) = −1.97, *p* = 0.51], Sadness: [t(220) = 1.28, *p* = 0.20], Fear: [t(220) = 1.33, *p* = 0.18], Anger: [t(220) = −1.00, *p* = 0.32]. Age difference for sadness [*F*(1, 221) = 4.41, *p* < 0.05].
H2	ANOVA, Independent sample t-test	Significant differences in sibling empathy based on birth order for fear, with second-born children showing higher empathy. Significant differences based on gender combination for anger, with two-girl families showing higher empathy than mixed-gender families.	Birth order for fear [t(220) = −3.29, *p* = 0.001]. Gender combination for anger [F(1, 221) = 4.45, *p* < 0.05].
H3	Pearson’s correlation	Positive association between general empathy and sibling empathy for happiness, sadness, fear, and anger.	Happiness: *r* = 0.29, *p* < 0.01; Sadness: *r* = 0.42, *p* < 0.01; Fear: *r* = 0.38, *p* < 0.01; Anger: *r* = 0.41, *p* < 0.01.
H4	Pearson’s correlation	Positive association between sibling empathy and sibling relationship quality.	Happiness: *r* = 0.25, *p* < 0.01; Sadness: *r* = 0.36, *p* < 0.01; Fear: *r* = 0.39, *p* < 0.01; Anger: *r* = 0.32, *p* < 0.01.
H5	SEM	Sibling empathy mediates the relationship between general empathy and sibling relationship quality.	Empathy significantly predicts sibling empathy (*B* = 0.73, *p* < 0.001). Empathy predicts sibling relationship (*B* = 0.08, *p* < 0.001). Sibling empathy predicts sibling relationship (*B* = 0.09, *p* < 0.001). Mediation effect: 43.36% of total effect, significant indirect effect.

Second, our results showed (refer to Table 11) no significant age and gender differences in sibling empathy, except for the age difference in response to “sadness.” This supports previous research suggesting that empathy in preschool children is largely independent of age and gender (Luo et al., 2019; Lam et al., 2012). The lack of age and gender differences in cognitive empathy may be due to its incomplete development in preschool children (Assing Hvidt et al., 2022; Rochat, 2023; Sommerlad et al., 2021). The development of sibling empathy in response to “sadness” underscores the importance of recognizing and understanding a sibling’s sadness for fostering sibling relationships. Empathy for “sadness” can induce prosocial behavior (Sallquist et al., 2009; Lockwood et al., 2014; Zahn-Waxler et al., 1995) but has also been linked to social anxiety in older age groups (Pittelkow et al., 2021; Tan et al., 2023).

Third, we found that second-born preschool children exhibited higher sibling empathy in response to “fear” compared to first-born children. This aligns with prior research indicating that both first-born and second-born children in two-child families display better empathy than only children (Qian et al., 2022; Zhang et al., 2019; Zhao and Yu, 2017). The transition from being an only child to having a sibling may explain why first-born children have lower sibling empathy, as younger siblings typically do not undergo this transition and are more likely to form attachments to older siblings (Kriss et al., 2018).

Fourth, sibling empathy in response to “anger” was higher in two-girl families compared to mixed-gender families. This finding is consistent with research showing that sisters are more empathetic and better at understanding each other’s feelings than brothers (Walęcka-Matyja, 2017; Buist et al., 2013; Gungordu and Hernandez-Reif, 2022). Same-gender siblings are more likely to model and observe each other’s behaviors, enhancing empathy (Kendler et al., 2014; Zhang et al., 2024; Slomkowski et al., 2001; Trim et al., 2006). Sibling anger can lead to conflict, and promoting perspective-taking in response to anger can be a constructive solution (Tucker et al., 2017; Dunn and Munn, 1986). Group empathy theory suggests that individuals are more likely to empathize with familiar or internal groups, such as same-gender siblings (Sirin et al., 2016; Sirin et al., 2017). Additionally, gender prejudice within families in China can affect sibling relationships through parenting styles (Hird, 2019).

Our results indicate that sibling empathy mediates the relationship between general empathy and sibling relationships among Chinese preschool children. Empathy is related to positive sibling relationships by increasing emotional understanding and connection between siblings.

The full mediation path observed in this study—where general empathy predicts sibling empathy, which in turn predicts sibling relationship quality—offers deeper theoretical significance when interpreted through both developmental and social-psychological frameworks. According to *Hoffman’s Theory of Empathy Development*, children gradually progress from global, affective responses (e.g., emotional contagion) toward more differentiated and context-specific forms of cognitive empathy. Our finding that general empathy significantly predicts sibling-directed empathy aligns with this trajectory: preschool children are beginning to apply their generalized empathic understanding to relationally meaningful contexts such as sibling interactions. This shift from abstract empathic concern to sibling-targeted responses may reflect the transition from early-stage affective empathy to more structured role-taking and emotional attribution skills.

Moreover, the second leg of the model—where sibling empathy enhances sibling relationship quality—is consistent with Group Empathy Theory, which emphasizes that empathy is more robust within emotionally salient in-groups. Siblings, who share daily routines, family roles, and often emotional histories, constitute a powerful in-group. The elevated impact of empathy within this dyad likely stems not only from shared identity but from repeated emotionally charged interactions that cultivate responsiveness. Thus, sibling empathy is not simply an application of general empathy; it may be a qualitatively distinct, socially constructed mechanism shaped by the emotional ecology of family life.

By integrating these perspectives, the model clarifies how emotional understanding developed in broader contexts is refined and activated within family systems. This supports the notion that sibling empathy is both developmentally emergent and contextually amplified—a key mechanism through which general socio-emotional capacities translate into close interpersonal relationship quality during early childhood.

Each link in the mediation model is noteworthy. The first part (empathy → sibling empathy) is supported by both theory and empirical research. Group empathy theory demonstrates that empathy varies across different groups, being stronger within familiar groups (Forgiarini et al., 2011; Gokcigdem, 2016; Kaseweter et al., 2012; Tarrant et al., 2009). Empirical studies also show that siblings’ empathetic concern predicts each other’s empathy (Jambon et al., 2019; Eisenberg et al., 2013). The second part (sibling empathy → sibling relationship) confirms that high levels of sibling empathy strengthen sibling relationships, consistent with the empathy-altruism hypothesis (Batson et al., 1991) and previous findings that empathy enhances social relationships (Zhang et al., 2019; Uzefovsky and Knafo-Noam, 2016; Guoying et al., 2021).

In this study, sibling empathy partially mediated the relationship between parental differential treatment and sibling relationships, accounting for 76.54% of the variance. This primary data provides evidence that sibling empathy mediates the relationship between general empathy and sibling relationships among Chinese preschool children in two-child families, revealing a new mechanism from the perspective of sibling empathy.

## 6 Theoretical and practical implications

The findings of this study have several significant theoretical and practical implications.

### 6.1 Theoretical implications

The study contributes to the existing body of literature on empathy development by providing new insights into sibling empathy among preschool children in China. Previous research has primarily focused on general empathy or sibling relationships separately, without specifically addressing the nuanced interactions between sibling empathy, general empathy, and sibling relationships (Jambon et al., 2019; Qian et al., 2022). By developing the Measurement of Sibling Empathy in Chinese Preschool Children (MSCP) and using it to explore these interactions, this study extends Hoffman’s Theory of Empathy Development to a new cultural and familial context (Hoffman, 1996).

The results support the notion that sibling empathy is a distinct construct influenced by both general empathy and sibling-specific dynamics. The finding that sibling empathy mediates the relationship between general empathy and sibling relationships highlights the importance of considering sibling-specific factors in empathy research. This aligns with group empathy theory, which suggests that empathy manifests differently based on group dynamics (McLoughlin and Over, 2017; Over, 2018).

Moreover, the age and birth order differences observed in this study provide empirical support for developmental and social learning theories. The higher levels of sibling empathy in second-born children, for example, suggest that younger siblings may develop empathy through social learning and observation of their older siblings (Kriss et al., 2018). This finding contributes to the understanding of how birth order and sibling interactions shape socio-emotional development, particularly in the context of China’s two-child policy (Chen et al., 2017).

### 6.2 Practical implications

Practically, the study offers valuable insights for parents, educators, and policymakers aiming to foster healthy sibling relationships and socio-emotional development in children. The positive association between sibling empathy and sibling relationship quality suggests that interventions designed to enhance sibling empathy could improve overall family dynamics. Given that sibling empathy mediates the relationship between general empathy and sibling relationships, programs that target empathy development in general can also have beneficial effects on sibling interactions.

Parents and educators can use the MSCP as a diagnostic and developmental tool to assess and support empathy growth in preschool children. The detailed scenarios and structured scoring of the MSCP offer a practical framework for identifying emotion-specific empathy deficits. For example, if a child demonstrates lower empathy in response to sadness, educators or parents might use emotion-based storytelling sessions where the child is guided to recognize the sibling’s emotional state, reflect on similar personal experiences, and practice comforting responses. These structured discussions can be incorporated into classroom activities or home routines to reinforce affective and cognitive empathy in real-life sibling contexts, ultimately enhancing prosocial behavior and emotional attunement.

Additionally, the study’s findings regarding the higher levels of sibling empathy in two-girl families compared to mixed-gender families suggest that gender dynamics play a crucial role in empathy development. This information can guide the creation of gender-sensitive interventions that address the unique needs of different sibling pairings, ultimately fostering more empathetic and supportive family environments (Buist et al., 2013; Gungordu and Hernandez-Reif, 2022).

Policymakers can also benefit from these findings by promoting family policies that support sibling interactions and emotional skill-building from early childhood. For instance, public initiatives that encourage family bonding activities, provide parenting workshops, or integrate socio-emotional curricula into preschools can help cultivate empathy in young children. Recognizing the cultural specificity of sibling relationships and emotional expression in China, such programs should be tailored to local values and parenting norms to ensure effectiveness and engagement (Solmeyer and McHale, 2017).

In summary, this study offers both theoretical advancements and practical applications by deepening the understanding of sibling empathy and its impact on sibling relationships among Chinese preschool children. The insights gained can inform future research, family interventions, and policy development aimed at enhancing socio-emotional development in early childhood.

## 7 Limitations and future research direction

This study aimed to explore the characteristics of sibling empathy and its relationship with general empathy and sibling relationships among Chinese preschool children. The findings indicated that: (1) cognitive empathy for the “happiness” emotion had developed to the level of attribution based on events, while other emotions remained at the level of no attribution or irrelevant attribution for one’s own emotion; (2) there were no significant age and gender differences in sibling empathy, except for the age difference in empathy for the “sadness” emotion; (3) second-born preschool children exhibited higher sibling empathy in response to the “fear” emotion compared to first-born children; (4) sibling empathy in response to the “anger” emotion was higher in two-girl families than in one-boy-one-girl families; and (5) sibling empathy significantly mediated the relationship between general empathy and sibling relationship quality.

However, several limitations of this study must be acknowledged. First, its cross-sectional design limits the ability to draw causal inferences. Longitudinal studies or experimental designs are needed to better understand the developmental trajectories and directional effects among general empathy, sibling empathy, and sibling relationship quality. Second, measurement issues arise from the primary reliance on maternal self-reports to assess sibling relationships. While parent reports offer useful perspectives, they may introduce social desirability bias or reflect subjective interpretations rather than direct observations. Prior research shows that different assessment methods (e.g., direct observation, multi-informant ratings) can produce divergent conclusions, particularly for the negative dimensions of sibling interaction such as jealousy or conflict (Dunn and Plomin, 1991). Future studies should triangulate data sources by incorporating observational methods, as well as self-reports from both children and other caregivers.

Third, the sample in this study—urban preschool children from two-child families in Zhejiang Province—represents a relatively homogeneous population. Most families were middle-class with well-educated parents, and the children shared similar cultural and socioeconomic contexts. This lack of diversity raises concerns about external validity and limits the generalizability of findings to broader populations, including children from rural areas, different socioeconomic strata, single-parent households, or ethnic minority communities. Additionally, cultural variables—such as familism, filial piety, or parental gender norms—may interact with empathy development and sibling dynamics in ways that were not fully captured in this study. Future research should adopt more diverse sampling strategies across regions and demographics, including cross-cultural comparisons, to assess the universality or cultural specificity of the observed effects.

Despite these limitations, the study makes several theoretical and practical contributions. Theoretically, while prior research has addressed general empathy or sibling relationships independently, this is the first study to conceptualize and empirically test sibling empathy as a mediating mechanism linking the two. By developing the Measurement of Sibling Empathy in Chinese Preschool Children (MSCP), this study also contributes a culturally adapted and psychometrically tested instrument that can support future empirical investigations.

Practically, the findings provide actionable insights for early childhood education and parenting. The results suggest that enhancing empathy in early childhood—particularly through sibling-focused interventions—could strengthen sibling bonds and promote positive socio-emotional outcomes. Emotion-specific training, especially in recognizing and responding to sadness or anger within sibling contexts, may be particularly effective. These insights can inform family education programs, teacher training, and policy initiatives focused on early childhood development in post–one-child policy China.

## Data Availability

The raw data supporting the conclusions of this article will be made available by the authors, without undue reservation.
